# Chronobiological activity of cysteinyl leukotriene receptor 1 during basal and induced autophagy in the ARPE-19 retinal pigment epithelial cell line

**DOI:** 10.18632/aging.203787

**Published:** 2021-12-17

**Authors:** Andreas Koller, Julia Preishuber-Pflügl, Christian Runge, Anja-Maria Ladek, Susanne Maria Brunner, Ludwig Aigner, Herbert Reitsamer, Andrea Trost

**Affiliations:** 1 Research Program for Experimental Ophthalmology, Department of Ophthalmology and Optometry, University Hospital of the Paracelsus Medical University, Salzburg 5020, Austria; 2 Institute of Molecular Regenerative Medicine, Spinal Cord Injury and Tissue Regeneration Center, Paracelsus Medical University, Salzburg 5020, Austria

**Keywords:** autophagy, CysLTR1, LC3B, lysosomal degradation, retinal pigment epithelial cells

## Abstract

Autophagy is an important cellular mechanism for maintaining cellular homeostasis, and its impairment correlates highly with age and age-related diseases. Retinal pigment epithelial (RPE) cells of the eye represent a crucial model for studying autophagy, as RPE functions and integrity are highly dependent on an efficient autophagic process. Cysteinyl leukotriene receptor 1 (CysLTR1) acts in immunoregulation and cellular stress responses and is a potential regulator of basal and adaptive autophagy. As basal autophagy is a dynamic process, the aim of this study was to define the role of CysLTR1 in autophagy regulation in a chronobiologic context using the ARPE-19 human RPE cell line.

Effects of CysLTR1 inhibition on basal autophagic activity were analyzed at inactive/low and high lysosomal degradation activity with the antagonists zafirlukast (ZTK) and montelukast (MTK) at a dosage of 100 nM for 3 hours. Abundances of the autophagy markers LC3-II and SQSTM1 and LC3B particles were analyzed in the absence and presence of lysosomal inhibitors using western blot analysis and immunofluorescence microscopy.

CysLTR1 antagonization revealed a biphasic effect of CysLTR1 on autophagosome formation and lysosomal degradation that depended on the autophagic activity of cells at treatment initiation. ZTK and MTK affected lysosomal degradation, but only ZTK regulated autophagosome formation. In addition, dexamethasone treatment and serum shock induced autophagy, which was repressed by CysLTR1 antagonization. As a newly identified autophagy modulator, CysLTR1 appears to be a key player in the chronobiological regulation of basal autophagy and adaptive autophagy in RPE cells.

## INTRODUCTION

Cysteinyl leukotrienes (CysLTs), LTC4, LTD4 and LTE4 are lipid mediators derived from arachidonic acid through the lipoxygenase pathway that are well known for their proinflammatory effects. CysLTs exert their functions via three known G protein-coupled receptors: cysteinyl leukotriene receptor 1 and 2 (CysLTR1 and CysLTR2) and 2-oxogulutarate receptor 1 (OXGR1) [1–4]. Along with immunoreactive functions, CysLTR1 and CysLTR2 play a role in stress-induced cell death, and CysLTR inhibitors increase cell survival after induction of the unfolded protein response (UPR), highlighting the interplay of the leukotriene system and UPR [5, 6]. Under basal conditions and moderate cellular stress, UPR pathways are essential regulators of the autophagic process through activation of transcription factor 4 (ATF4), ATF6 and X box-binding protein 1 (XBP1) [7–10]. However, prolonged endoplasmic reticulum (ER) stress and UPR activity lead to autophagy inhibition and cell death [11, 12].

Autophagy, which includes at least three forms, namely, macroautophagy, microautophagy and chaperone-mediated autophagy (CMA), is an intracellular process for degrading and recycling misfolded/long-lived proteins, lipid droplets, invading microorganisms and damaged organelles [13, 14]. The present study focuses on macroautophagy, herein called autophagy. Briefly, the autophagic process comprises five stages: 1) initiation, 2) nucleation of the isolation membrane (also named phagophore), 3) expansion of the isolation membrane and autophagosome formation with sequestered cellular cargo to be degraded, 4) fusion of the autophagosome and lysosome, and 5) degradation of the autolysosomal content [12, 15]. Impaired autophagy is a hallmark of diverse age-related diseases [16, 17], and defects in autophagic and lysosomal activities have been observed in retinal pigment epithelium (RPE) cells of patients with age-related macular degeneration (AMD) [18–21]. The RPE monolayer is a part of the retina located between the photoreceptor layer and Bruch’s membrane, forming the outer blood-retina barrier, and its functions are essential for retinal integrity maintenance [22, 23].

Recently, we demonstrated a correlation between autophagy-/UPR-related gene expression and arachidonate 5-lipoxygenase (ALOX5) gene expression in the ARPE-19 RPE cell line [24]. The enzyme ALOX5 metabolizes arachidonic acid, which results in the formation of leukotriene A4 (LTA4), the precursor of CysLTs [3]. CysLTR1 is expressed and localized to cytoskeletal microtubule structures in polarized ARPE-19 cells, and its inhibition by zafirlukast (ZTK) in the presence of lysosomal degradation inhibitors leads to increased levels of lipidated microtubule-associated protein 1 light chain 3 (LC3-II) [24], a marker of autophagic activity [25, 26]. Moreover, microtubule structures play a key role in autophagosome and lysosome transport and are essential for the fusion of autophagy-associated organelles [27–29]. Recently, two independent publications reported a contrary role of CysLTR1 in autophagy regulation, suggesting more complex involvement of CysLTR1 in these processes [30, 31].

Basal autophagy is chronobiologically regulated *in vivo*, as represented by time-dependent autophagosome formation and lysosomal degradation [32–35], and is essential for maintaining cellular homeostasis [36]. The chronobiological phase of autophagic activity is influenced by extrinsic and intrinsic mechanisms at systemic and cellular levels. In agreement, polarized ARPE-19 cells exhibit varying time-dependent LC3-II levels *in vitro* [24]. Although the autophagic activity of cells can be influenced *in vitro* by exogenous stimulation, such as the addition of fresh medium, the intrinsic self-regulating mechanism has a more decisive impact [26, 37].

As CysLTR1 inhibition in polarized ARPE-19 cells has revealed the biphasic potential of CysLTR1 to regulate expression of autophagy-related genes [24], the aim of this study was to characterize the possible chronobiological activity of CysLTR1 with regard to autophagosome formation and lysosomal degradation in polarized ARPE-19 cells using the CysLTR1 antagonists ZTK and montelukast (MTK).

## RESULTS

### ARPE-19 cells exhibit time-dependent lysosomal degradation activity

*In vivo*, basal LC3-II levels exhibit a time-dependent rhythm and correlate negatively with the activity of lysosomal degradation [32, 33]. Although *in vitro* culture conditions lack external zeitgeber, such as light or photoreceptor outer segment phagocytosis, which synchronize RPE cell activity *in vivo,* we recently observed intrinsic time-dependent autophagic activity in the RPE cell line ARPE-19 using the autophagy marker LC3-II [24]. To determine whether lysosomal degradation capacity is constant or varies over time, autophagic flux (activity of lysosomal degradation) was investigated at random time points during cell cultivation. Twenty-four hours prior to treatment, the culture medium was renewed. Monolayers were treated for 3 hours with a combination of the lysosomal inhibitors E64d and pepstatin A; cells were left untreated in time-matched control samples.

**Figure 1:**
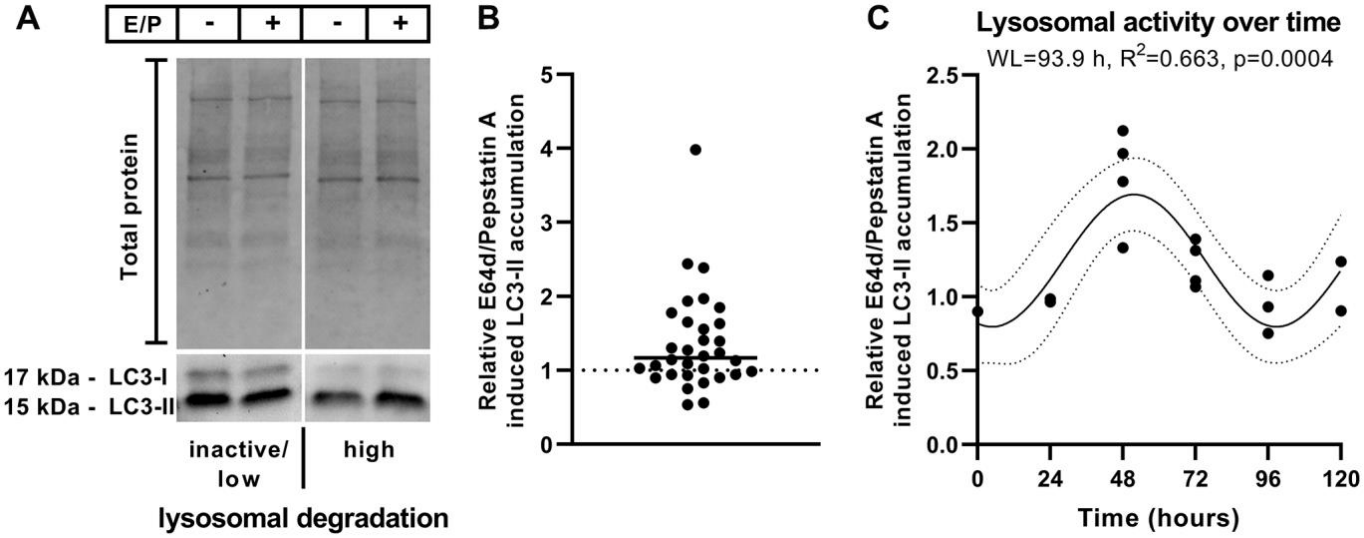
**Relative E64d/pepstatin A-induced LC3-II accumulation.** (**A**) Representative western blot of LC3-I and LC3-II levels in polarized ARPE-19 cells left untreated or treated for 3 hours with lysosomal inhibitors (10 μg/ml E64d + 10 μg/ml pepstatin A [E/P]). (**B**) Relative E64d/pepstatin A-induced LC3-II accumulation in different batches of polarized ARPE-19 cells treated on different days. *n* = 32 + median. (**C**) Overlay of relative E64d/pepstatin A-induced LC3-II accumulation within 72 hours of 4 different cell batches using the highest value as a reference point. Sine waves with nonzero baseline + confidence bands were generated with a comparison of fits (amplitude = 0 versus amplitude unconstrained). Abbreviation: WL: wavelength. Western blot images are cropped showing areas of marked primary antibody interaction only.

Lysosomal inhibition by E64d/pepstatin A treatment resulted in varying LC3-II accumulation ratios in diverse cell batches of polarized ARPE-19 cells at different times and days (accumulation ratio = LC3-II levels with lysosomal inhibitors/LC3-II levels without lysosomal inhibitors) (Figure 1A and 1B). A ratio of 1 indicates the absence of lysosomal degradation, suggesting that lysosomal inhibition has no impact on LC3-II levels [38]. In the present study, E64d/pepstatin A-induced LC3-II accumulation ratios between ~0.5 and 4 were observed under basal culture conditions (Figure 1B).

To assess whether the broad spectrum of measured lysosomal activity in ARPE-19 cells is due to spontaneous fluctuations or follows a time-dependent pattern, the lysosomal activity of cell batches was screened over 72 hours. The medium was exchanged every 24 hours to ensure similar medium conditions during treatments. Lysosomal activity over time of different cell batches was retrospectively overlaid using the highest value as a reference point (Figure 1C), and this overlaid lysosomal activity over time represents a theoretical period of 120 hours, peaking at 48 hours (Figure 1C). A sine wave with a nonzero baseline was generated using comparison of fits (amplitude = 0 versus amplitude unconstrained), which revealed an intrinsic time-dependent variation in lysosomal activity in polarized ARPE-19 cells (Figure 1C). In summary, the accumulation ratio is suitable for determining the autophagic activity status of ARPE-19 cells, enabling us to study the impact of CysLTR1 inhibition on basal autophagy *in vitro* in a chronobiological manner using cultured cells.

### Chronobiological regulation of autophagic activity by CysLTR1

Taking time-dependent basal autophagic activity into account, the E64d/pepstatin A-induced LC3-II accumulation ratio was determined for each sample (*n* = 32), and samples were then classified into two groups [38] representing inactive/low (accumulation ratio <1.2) and high (accumulation ratio ≥1.2) lysosomal degradation activity at treatment initiation. The median of the accumulation ratios (median = 1.17) (Figure 1B) and the sine wave baseline (= 1.23; Figure 1C) were used to assign each sample.

### CysLTR1 antagonization by zafirlukast affects autophagosome formation and lysosomal degradation

As ZTK treatment results in opposite regulation of autophagic genes [24], it has been hypothesized that the chronobiological activity of CysLTR1 has an effect on autophagy. Therefore, the effect of ZTK-induced CysLTR1 inhibition on LC3-II levels in the presence of lysosomal inhibitors (autophagosome formation) and lysosomal degradation (autophagic flux) was investigated in a time-dependent (lysosomal activity-dependent) manner. Polarized ARPE-19 cells were treated for 3 hours with ZTK in the absence or presence of lysosomal inhibitors.

#### LC3-I

In samples assigned to the inactive/low lysosomal degradation status group, LC3-I levels were unaffected by ZTK treatment, both in the absence and presence of lysosomal inhibitors (Supplementary Figure 1). However, lysosomal inhibitors alone led to a significant decrease (*p* = 0.020) in LC3-I levels in polarized ARPE-19 cells compared to the untreated control (Supplementary Figure 1). In samples assigned to the high lysosomal degradation status group, LC3-I levels were not regulated by either ZTK or lysosomal inhibition (Supplementary Figure 1).

#### LC3-II

**Figure 2:**
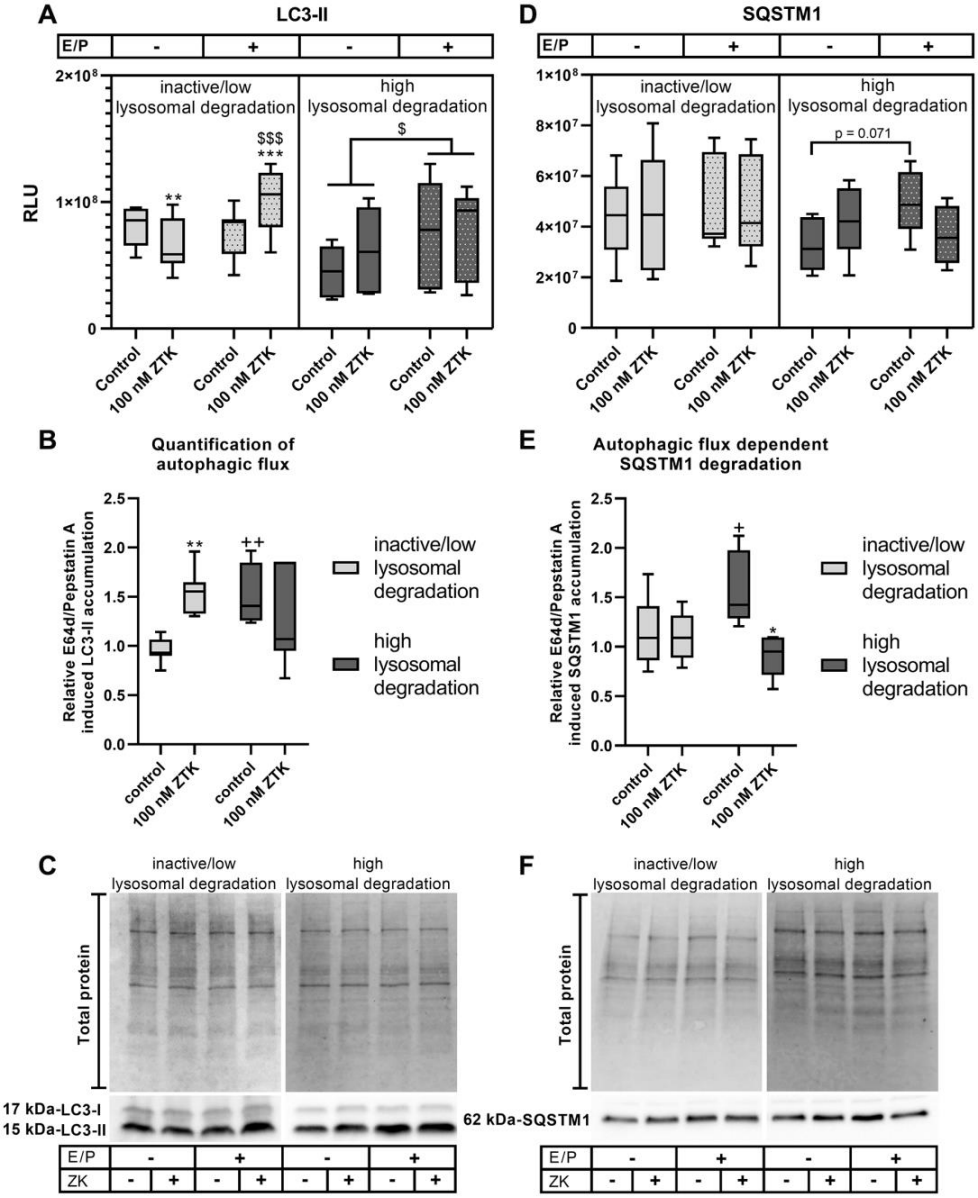
**LC3-II and SQSTM1 protein expression in polarized ARPE-19 cells treated with ZTK.** (**A**) Relative luminescence units (RLUs) of LC3-II normalized to the amount of total loaded protein in polarized ARPE-19 cells treated with 100 nM ZTK for 3 h in the absence and presence of lysosomal inhibitors E64d and pepstatin A (E/P). (**B**) Relative E64/pepstatin A-induced LC3-II accumulation in control and ZTK-treated polarized ARPE-19 cells. (**C**) Representative western blot analysis showing total protein loading, LC3-I and LC3-II expression in polarized ARPE-19 cells treated with ZTK in the absence and presence of lysosomal inhibitors E/P. (**D**) RLUs of SQSTM1 normalized to the amount of total loaded protein in polarized ARPE-19 cells treated with 100 nM ZTK for 3 h in the absence and presence of lysosomal inhibitors E/P. (**E**) Relative E64/pepstatin A-induced SQSTM1 accumulation in control and ZTK-treated polarized ARPE-19 cells. (**F**) Representative western blot analysis showing total protein loading and SQSTM1 expression in polarized ARPE-19 cells treated with ZTK in the absence and presence of lysosomal inhibitors E/P. Western blot images are cropped showing areas of marked primary antibody interaction only. Samples were grouped into inactive/low (autophagic flux of control <1.2) and high (autophagic flux of control ≥1.2) lysosomal degradation groups. Values are represented in box and whisker plot format (min to max); LC3-II: *n* = 7, SQSTM1: *n* = 6. The significance of differences (**A**, **D**) in LC3-II and SQSTM1 expression upon ZTK treatment was calculated for both groups via repeated measures two-way ANOVA (main factors: lysosomal inhibition (matched) and ZTK treatment (matched)) followed by a Tukey multiple comparison test. ^***^p < 0.001, ^**^p < 0.01 compared to the control, ^$$$^*p* < 0.001, ^$^*p* < 0.05 compared to the sample without lysosomal inhibition. The significance of differences in relative E64d/pepstatin A-induced (**B**, **E**) LC3-II and SQSTM1 accumulation after ZTK treatment was calculated by repeated measures two-way ANOVA (main factors: inactive/low and high lysosomal degradation and ZTK treatment (matched)) followed by a Sidak multiple comparison test. ^**^p < 0.01, ^*^p < 0.05 compared to control, ^++^*p* < 0.01, ^+^*p* < 0.05 compared to inactive/low lysosomal degradation sample.

In polarized ARPE-19 cells exhibiting inactive/low lysosomal degradation activity, ZTK led to a significant reduction in LC3-II levels in the absence of lysosomal inhibitors (*p* = 0.005) and to significant upregulation (*p* = 0.001) in the presence of lysosomal inhibitors compared to the control (Figure 2A, 2C). Accordingly, lysosomal degradation was induced by ZTK (*p* = 0.007) in the inactive/low lysosomal degradation group (Figure 2B). Lysosomal inhibitors alone had no effect on LC3-II levels (Figure 2A, 2C). During high lysosomal degradation activity, lysosomal inhibition resulted in significantly increased LC3-II levels compared to in the absence of E64d/pepstatin A (*p* = 0.015) (Figure 2A, 2C). Nevertheless, ZTK treatment had no effect on LC3-II levels in the absence or presence of lysosomal inhibitors (Figure 2A, 2C) or on autophagic flux (Figure 2B).

#### SQSTM1

Protein levels of ubiquitin-binding protein sequestosome-1 (SQSTM1), another marker frequently used to determine autophagic activity [38], were also investigated. Under inactive/low lysosomal degradation activity, neither ZTK treatment nor lysosomal inhibition influenced SQSTM1 levels (Figure 2D, 2F), resulting in unchanged autophagic flux-dependent SQSTM1 degradation (Figure 2E). In high lysosomal degradation status group samples, SQSTM1 protein levels exhibited a trend (*p* = 0.071) toward accumulation after lysosomal inhibition (Figure 2D, 2F). Although ZTK treatment had no effect on SQSTM1 levels in the absence or presence of lysosomal inhibitors (Figure 2D, 2F), it significantly reduced (*p* = 0.011) autophagic flux-dependent SQSTM1 degradation compared to the control (Figure 2E).

In summary, ZTK exhibited a clear effect on autophagy that correlated with lysosomal activity. This was also detected when relative E64d/pepstatin A-induced LC3-II accumulation in ZTK treatments compared to time-matched controls was examined over 72 hours, showing inverted lysosomal activity upon ZTK treatment compared to time-matched controls (Supplementary Figure 2).

### CysLTR1 antagonization by montelukast impacts lysosomal degradation

The effect of MTK was investigated to examine whether other CysLTR1 antagonists show properties similar to those of ZTK on autophagosome formation and autophagic flux. The experimental setup was identical to that of ZTK treatments.

#### LC3-I

In inactive/low lysosomal degradation group samples, LC3-I levels were significantly reduced by MTK treatment in the absence of lysosomal inhibitors (*p* = 0.003) but were unaffected in their presence (Supplementary Figure 3). In samples assigned to the high lysosomal degradation group, MTK treatment did not regulate LC3-I levels, independent of lysosomal inhibitors (Supplementary Figure 3).

#### LC3-II

**Figure 3:**
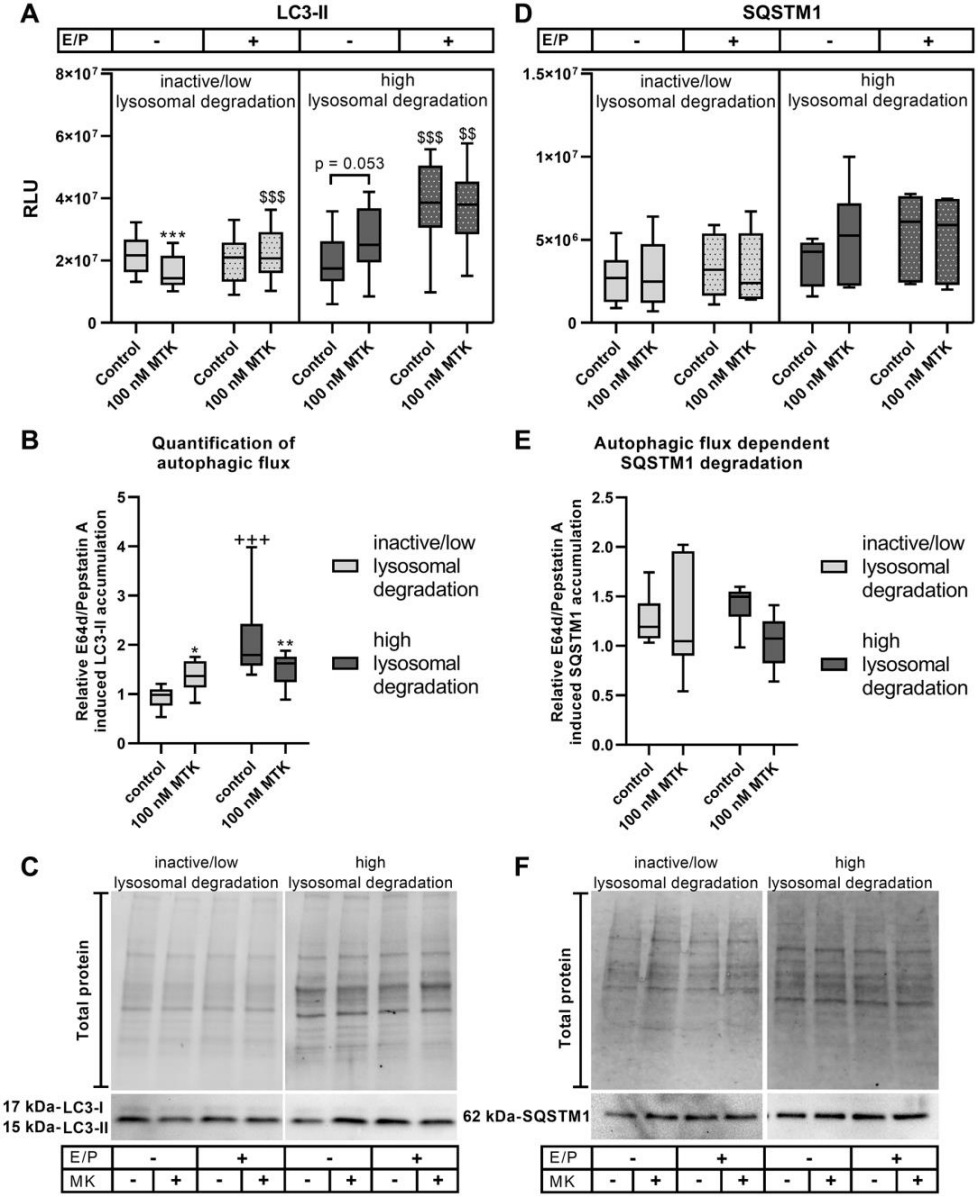
**LC3-II and SQSTM1 protein expression in polarized ARPE-19 cells treated with MTK.** (**A**) RLUs of LC3-II normalized to the amount of total loaded protein in polarized ARPE-19 cells treated with 100 nM MTK for 3 h in the absence and presence of lysosomal inhibitors E64d and pepstatin A (E/P). (**B**) Relative E64d/pepstatin A-induced LC3-II accumulation in control and MTK-treated polarized ARPE-19 cells. (**C**) Representative western blot analysis showing total protein loading, LC3-I and LC3-II expression in polarized ARPE-19 cells treated with MTK in the absence and presence of lysosomal inhibitors E/P. (**D**) RLUs of SQSTM1 normalized to the amount of total loaded protein in polarized ARPE-19 cells treated with 100 nM MTK for 3 h in the absence and presence of lysosomal inhibitors E/P. (**E**) Relative E64d/pepstatin A-induced SQSTM1 accumulation in control and MTK-treated polarized ARPE-19 cells. (**F**) Representative western blot analysis showing total protein loading and SQSTM1 expression in polarized ARPE-19 cells treated with MTK in the absence and presence of lysosomal inhibitors E/P. Western blot images are cropped showing areas of marked primary antibody interaction only. Samples were grouped into inactive/low (autophagic flux of control <1.2) and high (autophagic flux of control ≥1.2) lysosomal degradation groups. Values are represented in box and whisker plot format (min to max); LC3-II: *n* = 8–10, SQSTM1: *n* = 6. The significance of differences (**A**, **D**) in LC3-II and SQSTM1 expression upon MTK treatment was calculated for both groups by repeated measures two-way ANOVA (main factors: lysosomal inhibition (matched) and MTK treatment (matched)) followed by a Tukey multiple comparison test. ^***^p < 0.001 compared to the control, ^$$$^*p* < 0.001, ^$$^*p* < 0.01 compared to the sample without lysosomal inhibition. The significance of differences in relative E64d/pepstatin A-induced (**B**, **E**) LC3-II and SQSTM1 accumulation upon MTK treatment was calculated by repeated measures two-way ANOVA (main factors: inactive/low and high lysosomal degradation and MTK treatment (matched)) followed by a Sidak multiple comparison test. ^**^p < 0.01, ^*^p < 0.05 compared to control, ^+++^*p* < 0.001 compared to inactive/low lysosomal degradation sample.

Interestingly, during inactive/low lysosomal degradation activity, an effect of MTK treatment on LC3-II levels was only observed in the absence of lysosomal inhibitors, with significantly reduced LC3-II levels (*p* = 0.001), but not in the presence of lysosomal inhibitors (Figure 3A, 3C). Thus, relative E64d/pepstatin A-induced LC3-II accumulation was significantly increased by MTK (*p* = 0.046), indicating an induction of autophagic flux (Figure 3B). In polarized ARPE-19 cells exhibiting high lysosomal degradation activity, LC3-II levels were significantly increased (*p* = 0.001) upon lysosomal inhibition (Figure 3A). CysLTR1 inhibition by MTK did not affect LC3-II levels in the presence of lysosomal inhibitors but did display a trend toward increasing LC3-II in the absence of lysosomal inhibitors (*p* = 0.053) (Figure 3A, 3C). MTK treatment led to a significant reduction (*p* = 0.009) in relative E64d/pepstatin A-induced LC3-II levels in polarized ARPE-19 cells, suggesting that MTK inhibits autophagic flux (Figure 3B).

#### SQSTM1

SQSTM1 levels in the inactive/low lysosomal degradation group were unaffected by MTK treatment in the absence and presence of lysosomal inhibitors (Figure 3D, 3F), and MTK had no effect on autophagic flux-dependent SQSTM1 degradation (Figure 3E). In samples assigned to the high lysosomal degradation group, MTK treatment had no effect on SQSTM1 levels, independent of lysosomal inhibitors (Figure 3D, 3F) or autophagic flux-dependent SQSTM1 degradation (Figure 3E).

### CysLTR1 exhibits a biphasic effect on LC3B particle formation

**Figure 4:**
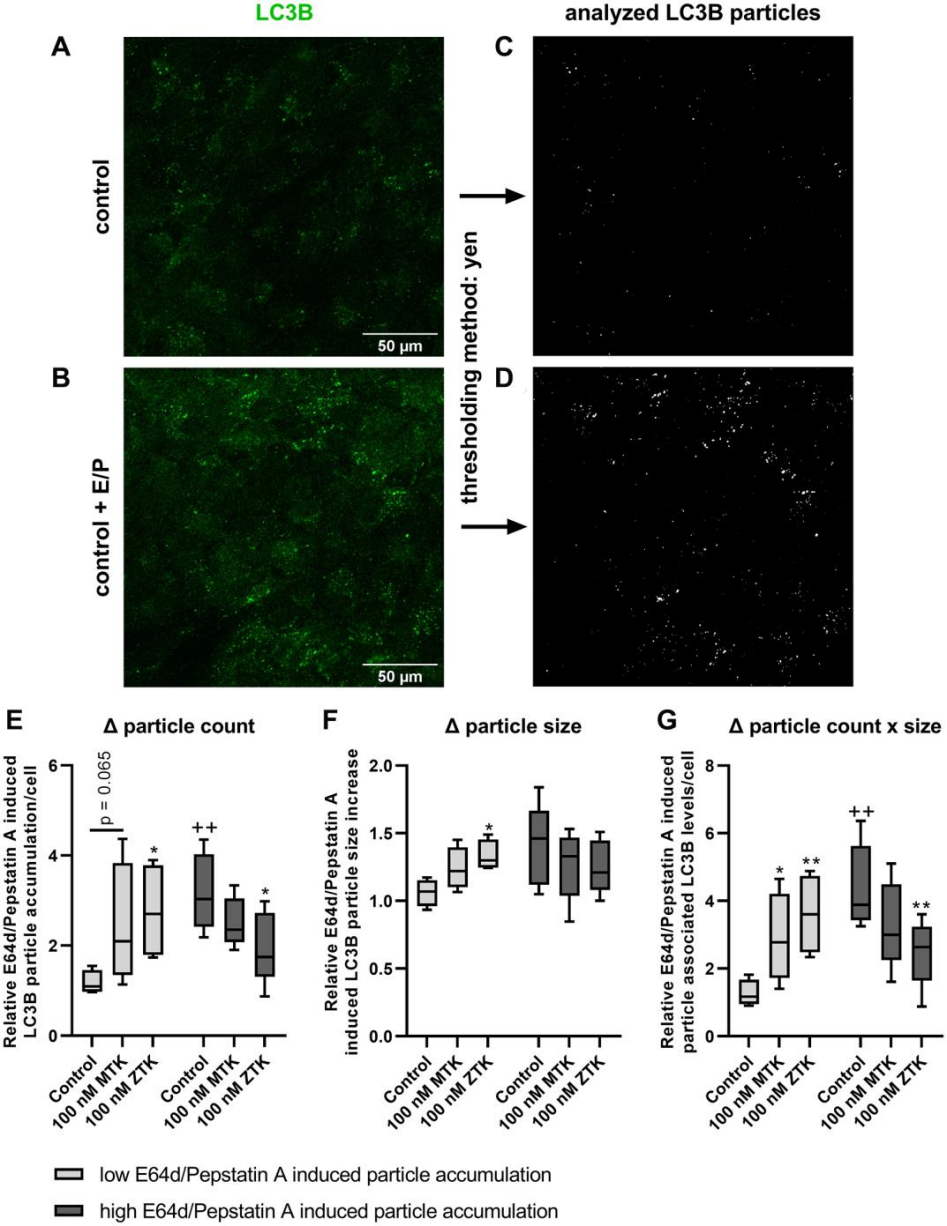
**LC3B particle analysis in polarized ARPE-19 cells upon MTK and ZTK treatment.** Representative IF images of polarized ARPE-19 cells in (**A**) the absence and (**B**) presence of lysosomal inhibitors E64d and pepstatin A for 3 h and the (**C**, **D**) corresponding LC3B particle count and size evaluation by ImageJ using the Yen thresholding method. Relative E64d/pepstatin A-induced LC3B particle (**E**) count accumulation, (**F**) size increase and (**G**) particle-associated LC3B levels/cell (particle count x size) of control, MTK- and ZTK-treated polarized ARPE-19 cells grouped in low and high E64d/pepstatin A-induced particle accumulation of control samples. Values are represented in box and whisker plot format (min to max); independent experiments: *n* = 4–5. The significance of differences in relative E64d/pepstatin A-induced LC3B particle count, size and count x size upon MTK and ZTK treatment was calculated by a repeated measures two-way ANOVA (main factors: low and high E64d/pepstatin A-induced particle accumulation and MTK/ZTK treatment (matched)) followed by a Dunnett multiple comparison test. ^**^p < 0.01, ^*^p < 0.05 compared to the control, ^++^*p* < 0.01 compared to the low E64d/pepstatin A-induced particle accumulation sample.

As polarized ARPE-19 cells showed a time-dependent lysosomal degradation capacity and corresponding CysLTR1 activity based on western blot analysis, changes in LC3B particles were investigated by immunofluorescence (IF) microscopy. Polarized ARPE-19 cells were treated with 100 nM MTK or 100 nM ZTK for 3 hours in the absence and presence of lysosomal inhibitors, and LC3B particles were quantified using ImageJ (thresholding method: Yen) (Figure 4A–4D). LC3B particles were analyzed for count per cell, size and count per cell x size (Supplementary Figure 4).

Similar to western blot analysis (Figures 1–3), lysosomal inhibitors exerted a variable effect on LC3B particle accumulation. Therefore, independent experiments were grouped into low (<2) and high (≥2) relative E64d/pepstatin A-induced particle accumulation per cell (ratio = particle count in samples with lysosomal inhibitors/particle count in samples without lysosomal inhibitors) in untreated control samples (Figure 4E). The control samples of defined groups significantly differed in particle count (*p* = 0.008) (Figure 4E) and count x size (*p* = 0.002) (Figure 4G) but not in particle size (Figure 4F).

In low E64d/pepstatin A-induced particle accumulation group samples, MTK treatment exhibited a trend (*p* = 0.065) of increased relative E64d/pepstatin A-induced LC3B particle accumulation per cell (Figure 4E), though the particle size remained unaffected by MTK (Figure 4F). Nevertheless, when both count and size were combined (count x size), MTK treatment significantly increased (*p* = 0.044) relative E64d/pepstatin A-induced particle-associated LC3B levels per cell (Figure 4G). Polarized ARPE-19 cells treated with ZTK also displayed a significant increase in relative E64d/pepstatin A-induced LC3B particle accumulation per cell (*p* = 0.020), size (*p* = 0.049) and particle-associated LC3B levels per cell (particle count x size, *p* = 0.005) (Figure 4E–4G).

In high E64d/pepstatin A-induced particle accumulation group samples, MTK treatment of polarized ARPE-19 cells did not affect relative E64d/pepstatin A-induced LC3B particle accumulation per cell, size or particle-associated LC3B levels per cell (particle count x size) (Figure 4E–4G). Interestingly, CysLTR1 inhibition by ZTK decreased relative E64d/pepstatin A-induced LC3B particle accumulation (*p* = 0.044) and particle-associated LC3B levels per cell (*p* = 0.010) but had no effect on LC3B particle size (Figure 4E–4G).

### Dexamethasone and serum shock synchronize cellular clock genes

Monolayers generated from a similar cell batch exhibit the same LC3-II levels at each time point investigated, and the effects of treatments on LC3-II levels are comparable to time-matched controls [25]. Nevertheless, monolayers originating from different cell batches show varying autophagic activity when compared to each other at each time point. As autophagy is a highly dynamic process, a rough classification of inactive/low and high lysosomal degradation activity only partially reflects the complex system of basal autophagy regulation and the involvement of CysLTR1. To achieve a potential reset of the autophagic system and thus to be able to compare different cell batches to each other at the same time, cells were treated with dexamethasone (DEX) or serum shock, two methods commonly used to synchronize the cellular clock *in vitro* [37]. Polarized ARPE-19 cells were treated with 1 μM DEX or exposed to serum shock (50% FBS) for 2 hours.

To verify whether DEX treatment and serum shock synchronizes the cellular clock in polarized ARPE-19 cells, expression of the clock genes Bmal1, Cry2, Per1 and Per2 was investigated by qPCR every 6–12 hours for up to 54 hours after synchronization (Supplementary Figure 5). DEX treatment clearly synchronized clock gene expression, with an expression rhythm (sine wave with nonzero baseline, wavelength [WL]) of approximately 28.3–32.1 hours (Supplementary Figure 5A–5D). Although serum shock also synchronized clock gene expression, except for Cry2, the R^2^ values of the corresponding sine waves were low (<0.31) (Supplementary Figure 5E–5H), representing weak cell synchronization.

### Serum shock but not dexamethasone treatment induces the unfolded protein response

To monitor whether synchronization treatments induce a cellular stress response because CysLTR1 activity is linked to UPR, expression of UPR-related transcription factors (ATF4, ATF6 and spliced XBP1 [XBP1s]) was analyzed at the mRNA level immediately after the 2-hour synchronization treatment. Although DEX had no significant impact on expression of UPR-related transcription factors, ATF4 showed a trend of reduced transcript levels after DEX treatment (*p* = 0.054, Supplementary Figure 6). Serum shock of polarized ARPE-19 cells significantly increased ATF4 mRNA levels (*p* = 0.019) but had no significant effect on ATF6 and XBP1s mRNA levels (Supplementary Figure 7A–7C).

As serum shock had a significant impact on mRNA expression of a single UPR transcription factor, intracellular protein levels of UPR transcription factors were further analyzed. Serum shock significantly induced ATF4 protein production in polarized ARPE-19 cells (*p* = 0.042; Supplementary Figure 7D, 7H). The ATF6 protein exists as a full-length protein (~90–100 kDa) in the ER and in its cleaved form (~50–60 kDa) as an active transcription factor [39, 40]. Serum shock had no significant effect on the levels of full-length ATF6 (Supplementary Figure 7E, 7I) but significantly reduced cleaved ATF6 levels in polarized ARPE-19 cells (*p* = 0.013; Supplementary Figure 7E, 7J). The XBP1s protein was barely detectable (Supplementary Figure 7F) and not regulated upon serum shock in polarized ARPE-19 cells (Supplementary Figure 7K). In summary, serum shock induces UPR and potentially affects CysLTR1 activity, as the leukotriene system plays a key role in the cellular stress response [6].

Therefore, the impact of serum shock on CysLTR1 protein levels was investigated, revealing no direct regulation after serum shock (Supplementary Figure 7G, 7L).

### Dexamethasone and serum shock induce autophagy in polarized ARPE-19 cells

**Figure 5:**
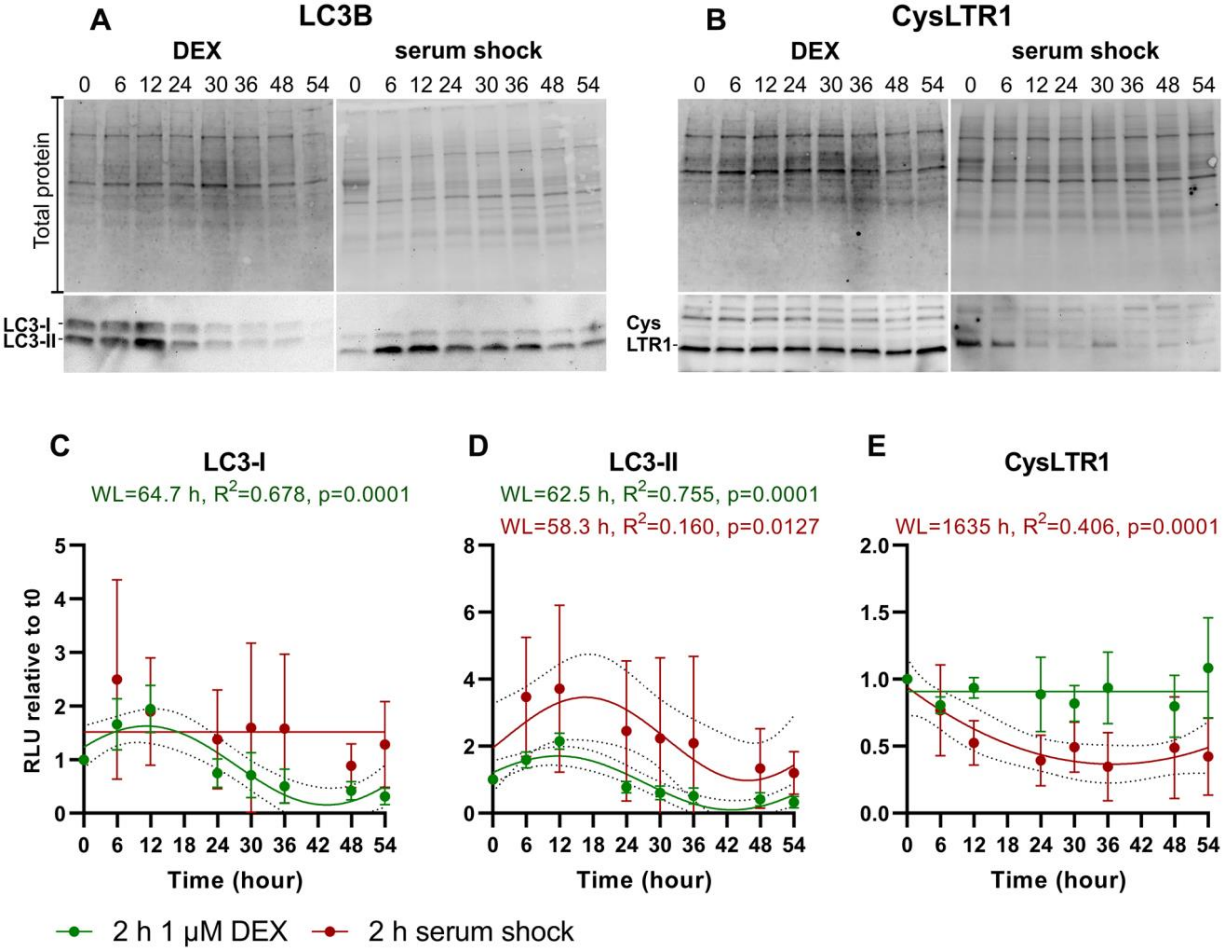
**Time series of LC3-I, LC3-II and CysLTR1 protein expression upon DEX treatment and serum shock in polarized ARPE-19 cells over a 54-hour period measured every 6 hours.** Representative western blot analysis showing total protein loading, (**A**) LC3-I and LC3-II and (**B**) CysLTR1 expression upon DEX treatment and serum shock in polarized ARPE-19 cells within 54 hours. RLU levels of (**C**) LC3-I, (**D**) LC3-II and (**E**) CysLTR1 upon DEX treatment (green) and serum shock (red) in polarized ARPE-19 cells within 54 hours. Values are represented as the mean ± SD; *n* = 3–5. Sine waves with nonzero baselines + confidence bands were generated with a comparison of fits (amplitude = 0 versus amplitude unconstrained). Abbreviation: WL: wavelength. Western blot images are cropped showing areas of marked primary antibody interaction only.

Isolated mRNA and protein samples were additionally analyzed for autophagy-related genes to investigate the impact of DEX and serum shock on autophagy synchronization. LC3-I and LC3-II protein levels were analyzed by western blot within a 54-hour period, and a sine wave with a nonzero baseline was generated using comparison of fits (amplitude = 0 versus amplitude unconstrained) (Figure 5A, 5C, 5D).

LC3-I levels were synchronized by DEX treatment (WL = 64.7 hours) but not by serum shock (Figure 5A, 5C). Moreover, LC3-II levels were synchronized by DEX treatment (WL = 62.5 hours) and serum shock (WL = 58.3 hours), peaking after 12 and 6–12 hours, respectively, and the lowest LC3-II levels were detected between 48–54 hours (Figure 5A, 5C, 5D). Interestingly, CysLTR1 levels remained constant after DEX treatment, whereas serum shock resulted in a reduction in CysLTR1 protein expression within 54 hours (Figure 5B, 5E).

Additionally, synchronization of autophagic genes, UPR transcription factors and ALOX5 was investigated at the mRNA level. DEX treatment synchronized the autophagy-related genes MAP1LC3B, BECN1, mTOR and the UPR transcription factor ATF6, showing a similar expression pattern within the 54-hour period upon DEX treatment (calculated WL = 72.3–80.4 hours) (Supplementary Figure 8A, 8C, 8D, 8F). Of note, the autophagy-related gene SQSTM1, the UPR transcription factor ATF4 and the lipoxygenase gene ALOX5 exhibited a concordant expression pattern (WL = 26.6–29.8 hours), which was different from the pattern observed for MAP1LC3B, BECN1, mTOR and ATF6 after DEX-induced synchronization (Supplementary Figure 8B, 8E, 8H). The UPR transcription factor XBP1s was downregulated upon DEX treatment (Supplementary Figure 8G). Serum shock induced only weak synchronization of BECN1, mTOR, ATF4, ATF6 or ALOX5, at which time only BECN1 and ATF6 exhibited a similar expression pattern within 54 hours (Supplementary Figure 8C–8F, 8H). However, mRNA levels of MAP1LC3B, SQSTM and XBP1s were not synchronized by serum shock (Supplementary Figure 8A, 8B, 8G).

### CysLTR1 antagonization inhibits dexamethasone and serum shock autophagy induction

To explore the impact of CysLTR1 signaling on LC3-II levels as a measure of autophagic activity following DEX treatment or serum shock, CysLTR1 was inhibited by MTK or ZTK treatment 6 hours prior to induction of the LC3-II peak. In addition, the effect of CysLTR1 inhibition on autophagy regulation by MTK and ZTK at 48 hours after DEX treatment and serum shock (when LC3-II levels reached a minimum) was investigated. Under both conditions, polarized ARPE-19 cells were treated with MTK or ZTK for 3 hours in the absence or presence of lysosomal inhibitors, and LC3-II levels were analyzed by western blot.

**Figure 6:**
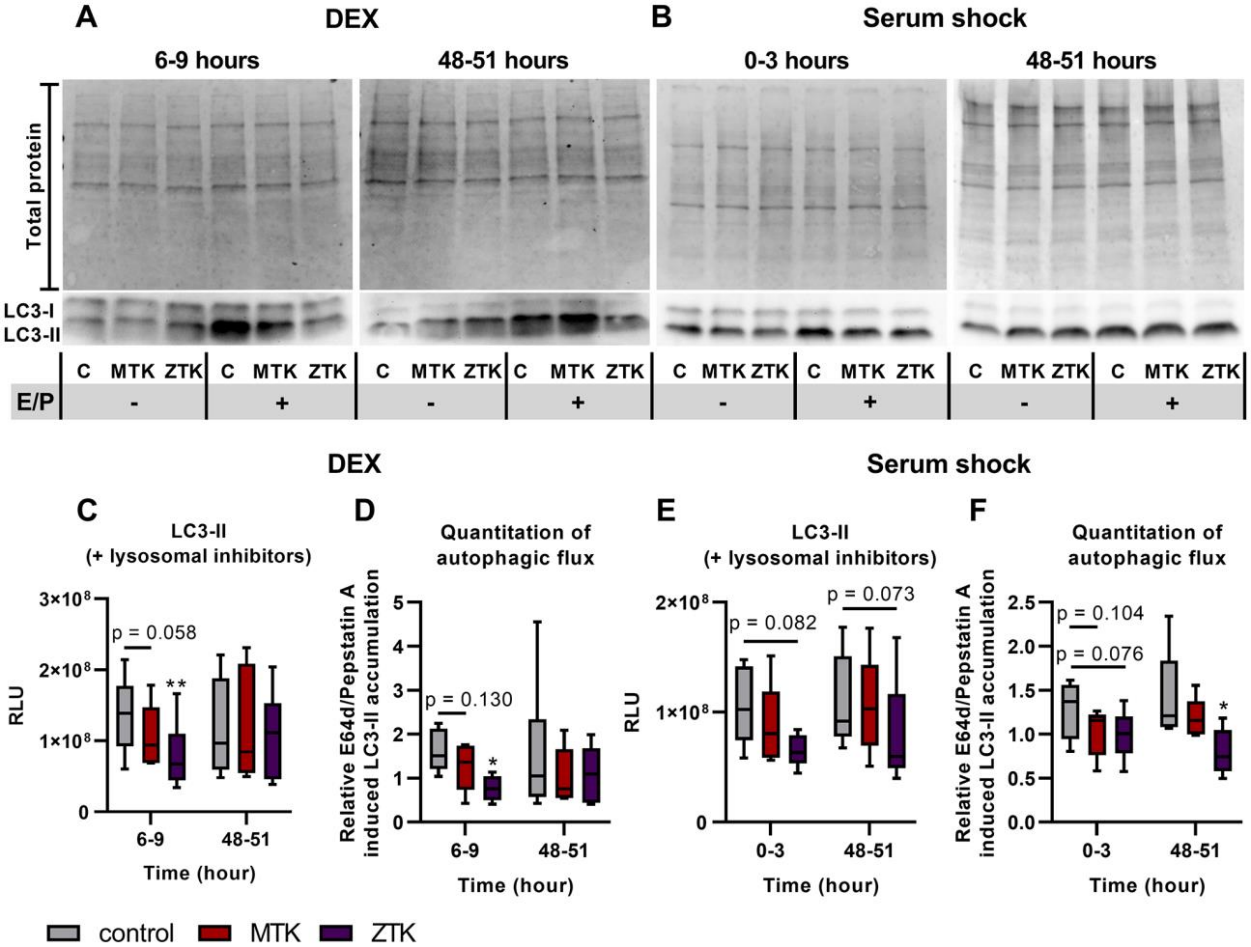
**LC3-I and LC3-II protein expression in polarized ARPE-19 cells treated with MTK or ZTK upon DEX treatment and serum shock.** Representative western blot analysis showing total protein loading and LC3-I and LC3-II expression at (**A**) 6 hours and 48 hours upon DEX treatment and (**B**) directly after and 48 hours upon serum shock in polarized ARPE-19 cells treated with 100 nM MTK or 100 nM ZTK in the absence and presence of lysosomal inhibitors E64d and pepstatin A (E/P). (**C**) RLU levels of LC3-II at 6 hours and 48 hours upon DEX treatment in the presence of lysosomal inhibitors for 3 hours in control samples (DMSO) and 100 nM MTK- or 100 nM ZTK-treated polarized ARPE-19 cells. (**D**) Relative E64d/pepstatin A-induced LC3-II accumulation in control, MTK- and ZTK-treated polarized ARPE-19 cells 6 and 48 hours upon DEX treatment. (**E**) RLU levels of LC3-II directly after and 48 hours upon serum shock in the presence of lysosomal inhibitors for 3 hours in control samples (DMSO) and 100 nM MTK- or 100 nM ZTK-treated polarized ARPE-19 cells. (**F**) Relative E64d/pepstatin A-induced LC3-II accumulation in control, MTK- and ZTK-treated polarized ARPE-19 cells directly after and 48 hours upon serum shock. Values are represented in box and whisker plot format (min to max); *n* = 5–6. The significance of differences in LC3-II expression upon MTK and ZTK treatment was calculated by repeated measures two-way ANOVA (main factors: time and treatment (matched)) followed by a Dunnett multiple comparison test. ^*^p < 0.05, ^**^p < 0.01. Western blot images are cropped showing areas of marked primary antibody interaction only.

Inhibition of CysLTR1 by ZTK at 6-9 hours after DEX treatment significantly reduced LC3-II levels in the presence of lysosomal inhibitors (*p* = 0.005), whereas MTK treatment resulted in a trend toward reduced LC3-II levels (*p* = 0.058) (Figure 6A, 6C). At 48 hours post DEX treatment, lysosomal activity in polarized ARPE-19 cells was inconsistent (inactive/low–high), and accordingly, CysLTR1 inhibition by MTK or ZTK led to up- or downregulation of LC3-II (in the presence of lysosomal inhibitors) (Figure 6C). Upon serum shock, LC3-II levels were not affected by MTK treatment (0-3 hours: *p* = 0.340, 48–51 hours: *p* = 0.790), but ZTK caused a trend toward downregulation of LC3-II levels in the presence of lysosomal inhibitors at 0–3 (*p* = 0.082) and 48–51 (*p* = 0.073) hours (Figure 6B, 6E).

Relative E64d/pepstatin A-induced LC3-II accumulation was significantly decreased by ZTK treatment at 6-9 hours after DEX treatment (*p* = 0.014), whereas CysLTR1 inhibition by MTK led to a reduced autophagic flux trend (*p* = 0.130) (Figure 6D). Additionally, MTK and ZTK treatments at 48 hours after DEX treatment had an inconsistent effect on autophagic flux, with no uniform regulation (Figure 6D). MTK treatment showed a trend toward reduced relative E64d/pepstatin A-induced LC3-II accumulation directly after serum shock (*p* = 0.104) but had no effect at 48 hours after serum shock (*p* = 0.304) (Figure 6F). Relative E64d/pepstatin A-induced LC3-II accumulation was significantly reduced by ZTK treatment at 48 hours post serum shock (*p* = 0.044), and a trend of reduction immediately after serum shock was also observed (*p* = 0.076) (Figure 6F).

## DISCUSSION

Basal autophagy is a dynamic process that exhibits time-dependent activity with respect to autophagosome formation, autophagosome-lysosome fusion and recycling of cellular waste within a repeated rhythm, which is clearly shown in mouse liver *in vivo* within 24 hours [32, 33]. Compared to the liver, levels of some autophagic proteins, such as LC3 or Atg9, are reported to peak twice within 24 hours in mammalian retinas, whereas other proteins, such as Beclin-1 and Atg7, peak just once a day [34, 35]. In the retina, rhythmic autophagic activity at the cellular level is modulated by extracellular and intracellular mechanisms, such as photoreceptor outer segment shedding from rods and cones and misfolded proteins or oxidative stress in RPE cells [32–35]. However, basal autophagy under *in vitro* conditions lacks external autophagy regulators, which are present *in vivo* and are mainly driven by intrinsic cellular processes. The main limitation of the present work is that studying basal autophagy under *in vitro* conditions does not consider external autophagy modulators, which are present *in vivo*. Furthermore, autophagy modulation by CysLTR1 still needs to be proven in primary RPE cells and other cell types in future studies.

The aim of the present study was to identify the time-dependent autophagic activity of CysLTR1 in polarized ARPE-19 cells by determining the status of autophagosome formation (changes to LC3-II levels in the presence of lysosomal inhibitors) and lysosomal degradation (autophagic flux).

ARPE-19 cells clearly exhibit intrinsically time-dependently regulated autophagic activity as LC3-II levels [24] and lysosomal degradation change over time. However, basal autophagy in polarized ARPE-19 cells under *in vitro* conditions is already affected by simple cell culture handling procedures, such as medium replacement [26], which hampers analysis of intrinsically regulated basal autophagy at the cellular level. Nevertheless, lysosomal activity in different cell batches of polarized ARPE-19 cells show a similar degradation capacity over time that may be slightly affected but is not completely abolished by exchanging the medium. In general, intrinsic factors contributing to a time-dependent basal pattern of autophagic activity might include accumulation of cellular waste, which triggers autophagy induction after exceeding a specific threshold. Furthermore, time-dependent autophagic activity might be affected by autophagic lysosome reformation (ALR), as ALR is required to restore the pool of lysosomes, which relies on mTOR1 reactivation and leads to autophagy inhibition [41].

In our *in vitro* setting, CysLTR1 inhibition by ZTK and MTK revealed biphasic activity of the receptor on autophagosome formation and lysosomal degradation, depending on the respective intracellular activity status of autophagy. When basal lysosomal degradation was low/inactive or low LC3B particle accumulation was observed after blocking lysosomal degradation, CysLTR1 antagonism increased autophagic activity by inducing autophagosome formation, as indicated by increased LC3-II protein levels and LC3B particle accumulation. Furthermore, increased lysosomal degradation activity was detected following CysLTR1 blockade at a low/inactive basal lysosomal degradation status. These findings indicate an inhibitory role of CysLTR1 on autophagic activity during low lysosomal degradation activity. Conversely, during high basal lysosomal degradation activity, CysLTR1 inhibition had a reducing effect on lysosomal degradation and LC3B particle accumulation after lysosomal blockade. This reveals a pro-autophagic activity of CysLTR1 during high lysosomal degradation activity. Regardless, when analyzing autophagosome formation upon ZTK treatment under high basal lysosomal degradation activity, LC3-II protein levels were inconsistent in individual treatment comparisons. In agreement, when the status of lysosomal activity was not taken into account but all samples were analyzed together, CysLTR1 inhibition by ZTK resulted in increased LC3-II levels in the presence of lysosomal inhibitors, as recently reported [24].

In contrast, under high basal autophagic flux conditions, ZTK treatment led to a constant reduction in LC3B particles by immunofluorescence (IF) microscopy, which was not observed in western blot analysis. This discrepancy may be explained by the difference in LC3B based on these methods: whole LC3-I and LC3-II levels are measured in western blot analysis, whereas IF analysis focuses only on accumulated high fluorescent LC3B particles while neglecting LC3B particles with low fluorescence intensity. Similarly, MTK treatment at high lysosomal activity significantly reduced lysosomal degradation, as shown by western blot analysis, but had no significant effect on LC3B particle accumulation. Nevertheless, both methods emphasize the chronobiological activity of CysLTR1 in autophagy regulation.

SQSTM1 (p62), an adaptor protein for selective autophagy of ubiquitinated substrates interacting with LC3, has been used as a marker of autophagic flux activity [38, 42]. Although autophagic flux (relative E64d/pepstatin A-induced LC3-II accumulation) was increased by CysLTR1 inhibition under low basal autophagic activity, SQSTM1 levels were unaffected, which probably indicates nonselective autophagy induction. During high basal autophagic flux activity, relative E64d/pepstatin A-induced SQSTM1 accumulation was reduced by CysLTR1 inhibition. These findings further highlight phase-dependent CysLTR1 activity in the cellular recycling system.

SQSTM1 also has a key role in the ubiquitin–proteasome system (UPS) and can be degraded by the proteasome [43]. Indeed, this should be considered, especially when using ZTK, as mean SQSTM1 protein levels were lower than those of the control in the presence of lysosomal inhibitors but were similar in their absence, even though the changes were not significant. These results suggest an additional role for CysLTR1 in UPS.

In summary, both CysLTR1 antagonists, namely, MTK and ZTK, regulate lysosomal activity, but only ZTK significantly affects LC3-II levels in the presence of lysosomal inhibitors. These data indicate that MTK and ZTK differentially disrupt CysLTR1 signaling properties or protein-protein interactions.

As microtubules are essential for autophagosome and lysosome transport to the perinuclear area, where the organelles fuse and the content is recycled, localization of membrane-bound CysLTR1 at microtubule structures [24] may play an important role in the time-dependent autophagosome formation and transport observed in our study [27–29]. Nonetheless, we did not investigate whether the cellular localization of CysLTR1 varies in accordance with its time-dependent activity on autophagic modulation. Furthermore, the interplay of CysLTR1 with the motor proteins dynein and kinesin located on microtubules in the context of autophagosome/lysosome transport should be explored in future studies.

Overall, synchronization of cell batches would be beneficial for examining basal autophagy of *in vitro* cultured ARPE-19 cells at defined time points in relation to intrinsic cellular clock. As DEX treatment and serum shock are reported to synchronize the cellular clock system [37], both methods were used in the present study to reset the cellular clock and, as a consequence, reset the autophagic process. DEX treatment resulted in synchronization of expression patterns of clock genes, though the effect of serum shock was less apparent. Furthermore, DEX treatment led to synchronization of autophagy-, UPR-related and ALOX5 gene expression. However, the clock genes displayed an expression rhythm of approximately 30 hours, whereas the autophagic genes (MAP1LC3B, BECN1 and mTOR) and UPR transcription factor ATF6 exhibited a calculated WL between ~70–80 hours and therefore seemed to be uncoupled from the cellular clock system. Nevertheless, the autophagy/UPS-related gene SQSTM1 and UPR-related genes ATF4 and ALOX5 were synchronized in a time pattern similar to that of the clock gene Per2. This observation indicates a complex relationship between the cellular clock, autophagy/UPS, UPR and leukotriene activity.

Although these synchronization methods have different mechanistic impacts on the cellular system [37], both seem to induce autophagy, as LC3-II and lysosomal degradation increased after DEX treatment and serum shock. Therefore, synchronizing autophagy via commonly used synchronization methods such as DEX or serum shock induces an adaptive autophagy response rather than synchronization of basal autophagic activity. Nevertheless, CysLTR1 appears to be involved in DEX- and serum shock-induced autophagy induction, as revealed by decreased LC3-II levels upon receptor antagonization in the presence of lysosomal inhibitors and a reduction in autophagic flux, respectively. These findings are similar to the results observed after CysLTR1 inhibition during high basal lysosomal activity, as autophagy was repressed in both cases, possibly through the same mechanism.

However, in polarized ARPE-19 cells, serum shock induced UPR and, more precisely, triggered ATF4 protein expression, which was not observed in DEX-treated cells. Increased ATF4 activity might modulate the autophagic response upon serum shock [7], which may explain the weak synchronization of the clock, autophagy and UPR genes. These data suggest a general pro-autophagic role of CysLTR1 in acute autophagy induction, especially as CysLTR1 inhibition following H_2_O_2_-induced autophagy leads to similar results [24]. Interestingly, DEX treatment had no impact on CysLTR1 expression levels, but serum shock reduced CysLTR1 within 54 hours after autophagy induction. These findings suggest that CysLTR1 regulation may be associated with the ER stress response, especially ATF4 activity, induced by serum shock because ER stress induction by brefeldin A treatment similarly attenuates CysLTR1 protein levels in the UPR cell-protective phase [6]. Regardless, further studies are needed to elucidate the mechanism of CysLTR1 regulation after serum shock.

In summary, CysLTR1 exhibits chronobiological activity regarding autophagy regulation in polarized ARPE-19 cells by supporting or inhibiting autophagosome formation and lysosomal degradation. Furthermore, DEX and serum shock, which are primarily used to achieve synchronization of autophagic processes, induce autophagy, and serum shock also activates UPR via ATF4 signaling. CysLTR1 was shown to be involved in autophagy induction upon DEX treatment and serum shock, as CysLTR1 inhibition reduced LC3-II levels (in the presence of lysosomal inhibitors) and autophagic flux. This study provides important data as a basis to investigate CysLTR1 as an RPE-specific or general autophagy modulator and to clarify the complex mechanism by which CysLTR1 affects the autophagic process in *in vitro* and *in vivo* experiments.

The role of malfunctioning basal autophagy and impaired adaptive autophagy upon additional cellular stress in aging and age-related diseases is the focus of gerontology research [17, 44]. Therefore, it is essential to investigate key regulatory players of autophagy to understand the underlying mechanisms contributing to the development of multifactorial diseases. Such insight is necessary for understanding complex autophagy regulation and developing strategies to ameliorate disease manifestation.

## MATERIALS AND METHODS

### Cell lines

The human RPE cell line ARPE-19 (male origin, obtained from ATCC, VA, USA) was cultivated and polarized as recently described under standardized normoxic conditions in a 37°C humidified incubator with 5% CO_2_ [24]. The cells were expanded in DMEM/F12 (ATCC) medium containing 10% fetal bovine serum (FBS, Thermo Fisher Scientific, MA, USA). For cell polarization, ARPE-19 cells were grown to 100% confluence and then cultured in medium containing 2% FBS for 7–12 days. All cell polarizations and treatments were performed in 12-well plates (Sigma-Aldrich, MO, USA).

### Cell treatment/CysLTR1 inhibition

Polarized ARPE-19 cells were left untreated (DMSO = 0.001%) or treated with 100 nM montelukast (MTK, Selleckchem, TX, USA) or zafirlukast (ZTK, Selleckchem) for 3 hours (DMSO concentration ≤ 0.001%). Twenty-four hours before treatment, the culture medium was renewed. For western blot analysis, CysLTR1 inhibition was performed in the absence and presence of 10 μg/ml E64d (Selleckchem) and 10 μg/ml pepstatin A (Selleckchem) (DMSO concentration ≤ 0.2%). All treated cell samples were compared to a time-matched vehicle-treated control sample with or without lysosomal inhibitors.

For synchronization, cells were treated with 1 μM DEX for 2 hours or subjected to serum shock by using 50% FBS in DMEM/F12 medium.

### Western blot analysis

Whole-protein isolations were performed using RIPA lysis buffer (Santa Cruz Biotechnology, TX, USA). Proteins were separated by SDS-PAGE using TGX stain-free gels (AnykD and 4–15% gels, Bio-Rad) and transferred to a PVDF membrane (Bio-Rad) by wet electroblotting (Bio-Rad). Total loaded protein was used for normalization of target protein expression (Image Lab 6.0.1, Bio-Rad). The PVDF membranes were blocked for 15 minutes with EveryBlot (Bio-Rad) at room temperature, followed by incubation overnight with a recombinant anti-LC3B antibody [EPR18709] (1:1000 at 4°C, ab192890, Abcam, UK), anti-CysLT1 antibody (1:500 at RT, ab151484, Abcam), anti-ATF4 antibody (1:1000 at RT, Cell Signaling, MA, USA), anti-ATF6 antibody (1:1000 at RT, Cell Signaling) or anti-XBP1s antibody (1:500 at RT, BioLegend, CA, USA) diluted in EveryBlot. Antibodies against LC3B, CysLTR1, ATF4 and ATF6 were labeled using an anti-rabbit antibody conjugated to HRP (Agilent, CA, USA), and anti-XBP1s was labeled using an anti-mouse antibody conjugated to HRP (Agilent) diluted in EveryBlot. The antibodies were visualized using Clarity Western ECL Substrate (Bio-Rad) and the ChemiDoc XRS + system (Bio-Rad).

### Immunofluorescence microscopy

ARPE-19 cells were polarized in 8-well chamber slides (Corning, NY, USA), and IF was performed as previously described [24, 45]. LC3B particles were visualized using a recombinant anti-LC3B antibody [EPR18709] (1:200, ab192890, Abcam, UK) and a donkey anti-rabbit antibody conjugated to Alexa Fluor 488 (1:1000, A-21206, Thermo Fisher Scientific). For secondary antibody-only controls, incubation was performed in the absence of primary antibodies, after which the samples were exposed to fluorescence-labeled secondary antibodies.

### Documentation

A confocal laser-scanning unit (Axio Observer Z1 attached to LSM710, Zeiss, Germany; 40 × oil immersion objective lens, numerical aperture 1.30, Zeiss) was used to document IF images. The single optical section mode was used for image acquisition with appropriate filter settings for Alexa Fluor 488 (495 nm excitation) and 4′, 6-diamidino-2-phenylindole (DAPI) (345 nm excitation). Five IF images for each treatment were randomly captured within the chamber slide. The LC3B particle count and size were evaluated using ImageJ 1.53c. Alexa Fluor 488 intensity for each experiment was adjusted to reach a similar LC3B particle count in the untreated control sample of all independent experiments using the Yen thresholding method (color threshold).

### RNA isolation/cDNA synthesis/qPCR

RNA from polarized ARPE-19 cells was isolated using High Pure RNA Isolation Kit (Roche, Switzerland) following the manufacturer’s instructions. cDNA was synthesized using iScript Kit (Bio-Rad) according to the manufacturer’s protocol. BRYT Green dye-based GoTaq qPCR Master Mix (Promega, WI, USA) was employed for quantitative PCR (qPCR) with the CFX96 system (Bio-Rad). The specific primers used for qPCR are listed in Table 1. Expression data were normalized to the reference gene GUSB by using CFX Manager 3.1 Software (Bio-Rad). GUSB exhibited stable expression and was not affected by intrinsic metabolism [24].

**Table 1:** Primer sequences for qPCR.

	**Forward 5′–3′**	**Reverse 5′–3′**
**GUSB**	AGCGAGTATGGAGCAGAAAC	TGATCCAGACCCAGATGGTA
**Bmal1**	ACAGAACACCAAGGAAGGATAAA	ACATTGCGTTGCATGTTGGT
**Cry2**	GGTTCCCCACTGAAGGACTTG	TCGGGGTCTCTCATAGTTGG
**Per1**	GTCCGTCTTCTGCCGTATCA	GATGCGCTCTGCAATCAGC
**Per2**	CACAGTTTCACCTCCCCGTA	CTTTTCCGGACACTGACACG
**MAP1LC3B**	CGTCGGAGAAGACCTTCAAG	CTGCTTCTCACCCTTGTATCG
**SQSTM1**	TGAAACACGGACACTTCGG	TCAGGAAATTCACACTCGGATC
**BECN1**	ACGAGTGTCAGAACTACAAACG	TTTCCACATCTTCCAGCTCC
**mTOR**	TTCGTGCCTGTCTGATTCTC	ATCCCGATTCATGCCCTTC
**ALOX5**	CATCGAGTTCCCCTGCTACC	ACGTCGGTGTTGCTTGAGAA
**ATF4**	ATGGGTTCTCCAGCGACAAG	GGCATCCAAGTCGAACTCCT
**ATF6**	GCCGCCGTCCCAGATATTA	CCGAGTTCAGCAAAGAGAGC
**XBP1s**	GCTGAGTCCGCAGCAGGT	CTGGGTCCAAGTTGTCCAGAAT

### Statistical analysis

Statistical analysis was performed using GraphPad Prism 9.0.0 (GraphPad Software, Inc., CA, USA). The statistical tests performed are described in each corresponding figure legend. Values were controlled for a normal distribution (Shapiro-Wilk test) to perform a parametric or nonparametric test. A *p*-value of <0.05 was considered statistically significant.

## Supplementary Materials

Supplementary Figures
