# Supplementary material for: Chronobiological activity of cysteinyl leukotriene receptor 1 during basal and induced autophagy in the ARPE-19 retinal pigment epithelial cell line
Source: Aging (Albany NY). 2021 Dec 17;13(24):25670–93. doi: 10.18632/aging.203787 (PMC8751616; doi:10.18632/aging.203787)
Supplement: Supplementary Figures [file aging-13-203787-s001.pdf]

SUPPLEMENTARY FIGURES

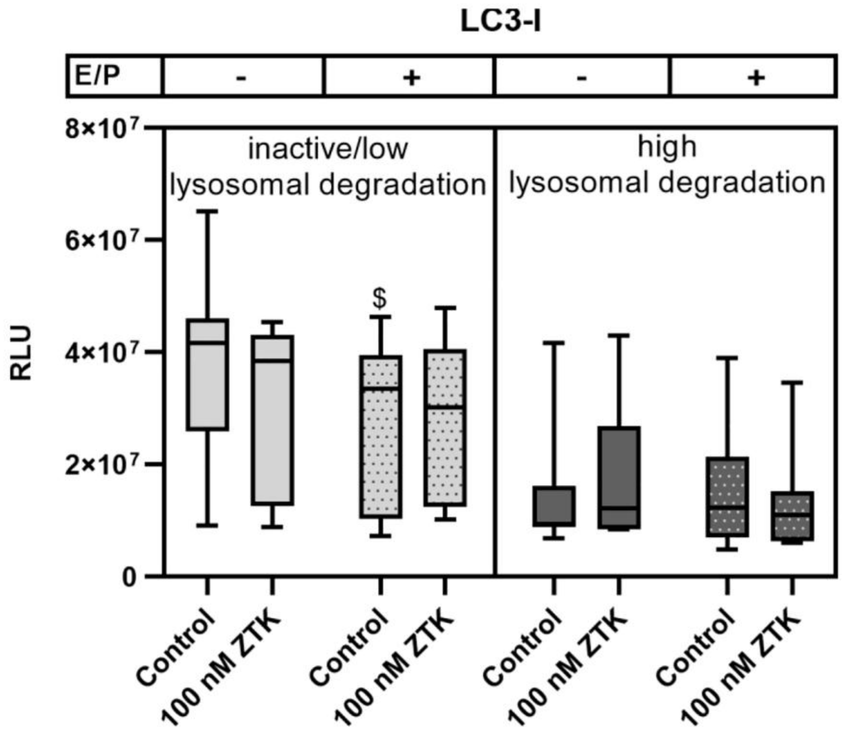

Supplementary Figure 1. LC3-I expression in polarized ARPE-19 cells treated with ZTK. RLUs of LC3-I normalized to the amount of total loaded protein in polarized ARPE-19 cells treated with 100 nM ZTK for 3 hours in the absence and presence of lysosomal inhibitors E64d and pepstatin A (E/P). Samples were grouped into inactive/low (autophagic flux of control <1.2) and high (autophagic flux of control ≥ 1.2) lysosomal degradation groups. Values are represented in box and whisker plot format (min to max); *n* = 7/7. The significance of differences in LC3-I expression upon ZTK treatment in both groups was calculated by repeated measures two-way ANOVA (main factors: lysosomal inhibition (matched) and ZTK treatment (matched)) followed by a Tukey multiple comparison test. $^{\$}p < 0.05$ compared to the sample without lysosomal inhibition.

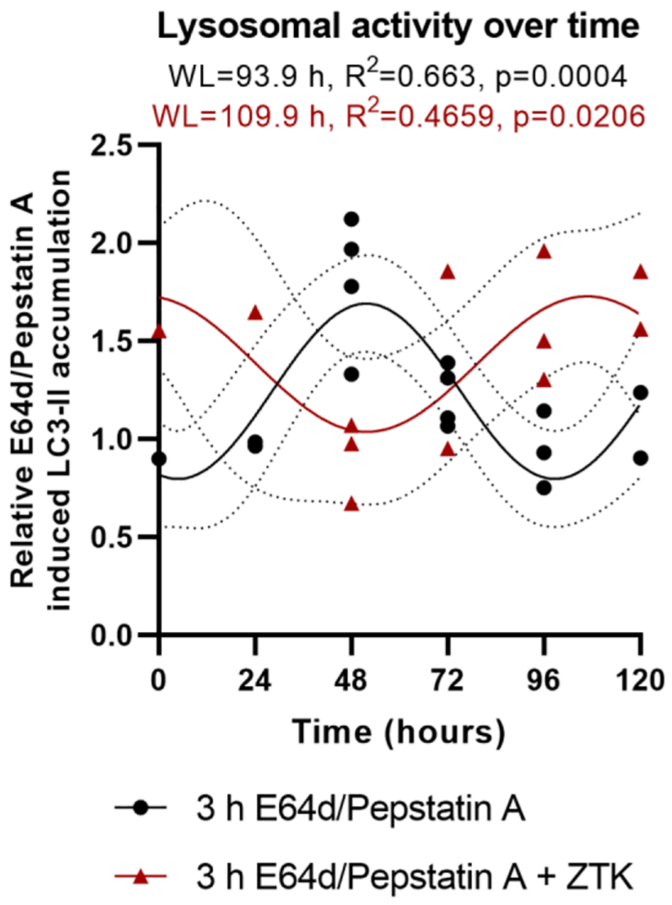

Supplementary Figure 2. Lysosomal activity over time. Overlay of relative E64d/pepstatin A-induced LC3-II accumulation of controls and the corresponding ZTK treatments within 72 hours of 3–4 different cell batches using the highest value of control samples as reference point. Sine waves with nonzero baseline + confidence bands were generated with comparison of fits (amplitude = 0 versus amplitude unconstrained). Abbreviation: WL: wavelength.

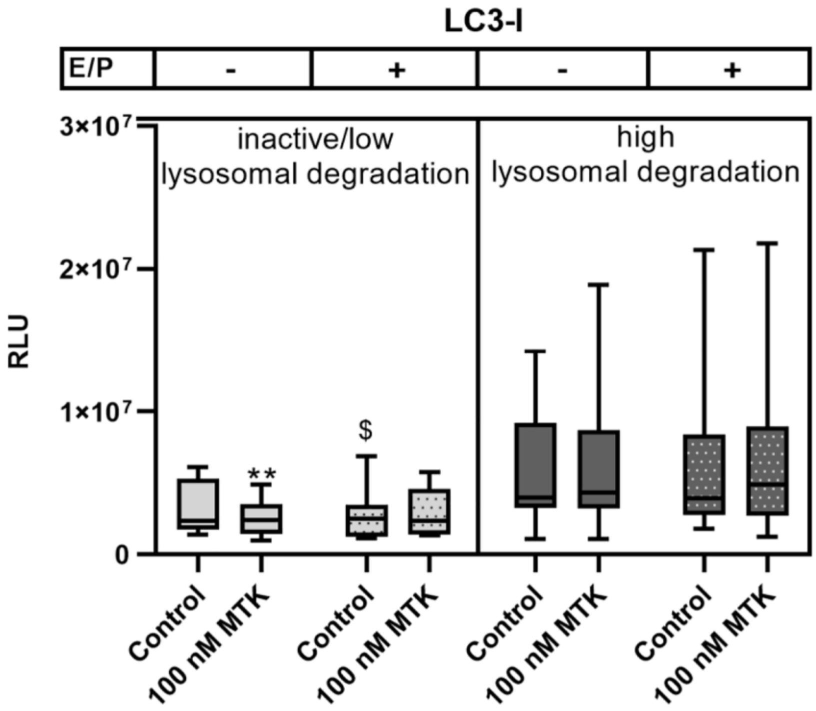

Supplementary Figure 3. LC3-I protein expression in polarized ARPE-19 cells treated with MTK. RLUs of LC3-I normalized to the amount of total loaded protein in polarized ARPE-19 cells treated with 100 nM MTK for 3 hours in the absence and presence of lysosomal inhibitors E64d and pepstatin A (E/P). Samples were grouped into inactive/low (autophagic flux of control <1.2) and high (autophagic flux of control ≥ 1.2) lysosomal degradation groups. Values are represented in box and whisker plot format (min to max); *n* = 10/8. The significance of differences in LC3-I expression upon MTK treatment was calculated in both groups by repeated measures two-way ANOVA (main factors: lysosomal inhibition (matched) and MTK treatment (matched)) followed by a Tukey multiple comparison test. $^{**}p < 0.01$ compared to the control, $^{\$}p < 0.05$ compared to the sample without lysosomal inhibition.

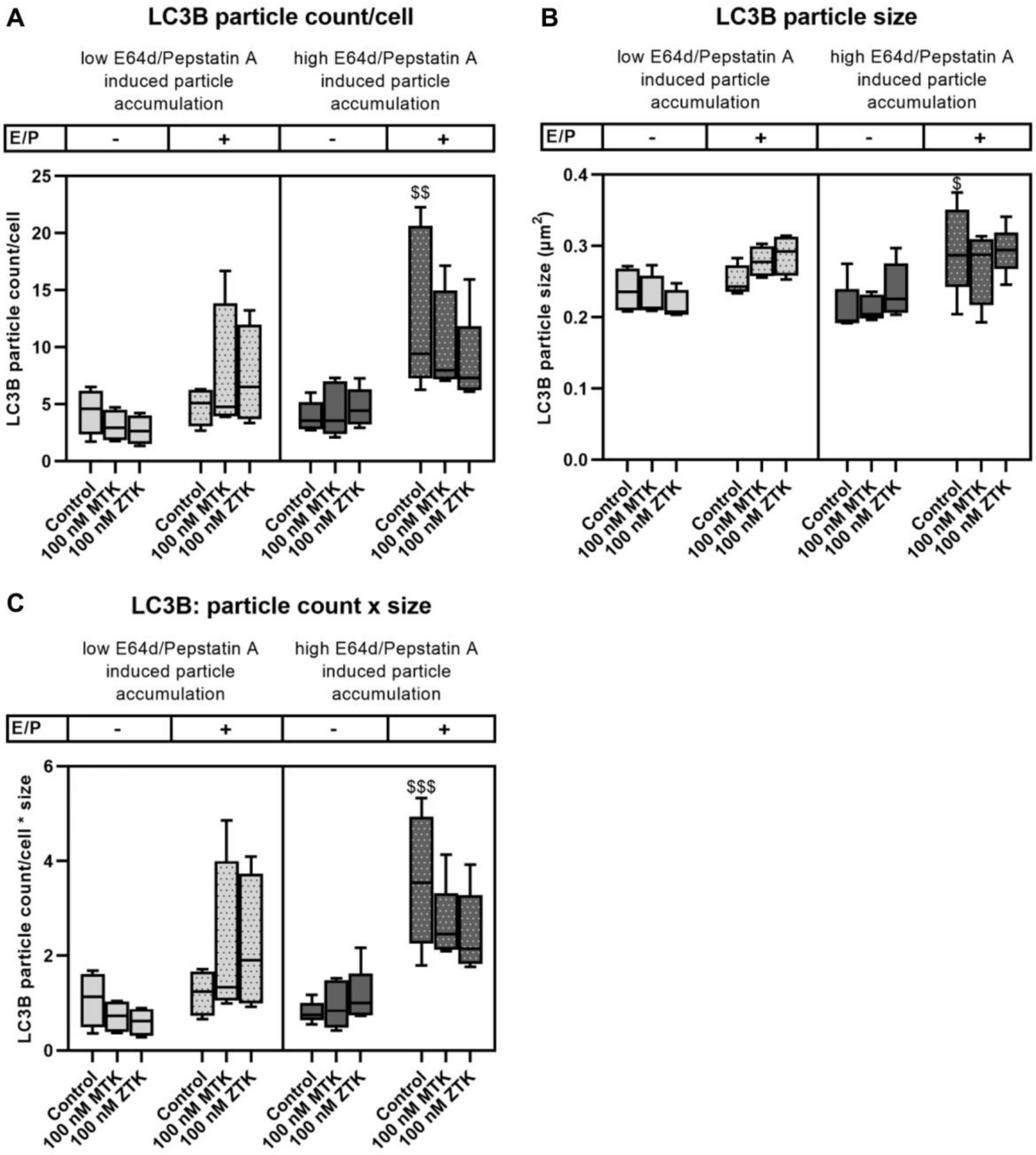

Supplementary Figure 4. LC3B particle count and size in ARPE-19 cells treated with MTK and ZTK. LC3B in polarized ARPE-19 cells treated with 100 nM MTK or 100 nM ZTK for 3 hours in the absence and presence of lysosomal inhibitors E64d and pepstatin A (E/P) was visualized using IF microscopy. LC3B particle (**A**) count and (**B**) size were analyzed using ImageJ software (thresholding method: Yen). (**C**) Relative particle-associated LC3B levels were calculated by multiplying particle counts per cell by particle size. Samples were grouped into no/low (relative particle increase <2) and high (relative particle increase ≥2) E64d/pepstatin A-induced particle accumulation. Values are represented in box and whisker plot format (min to max); *n* = 4/5. The significance of differences in LC3B particle count and size upon MTK and ZTK treatment was calculated by repeated measures three-way ANOVA (main factors: inactive/low and high lysosomal degradation, lysosomal inhibition (matched) and MTK/ZTK treatment (matched)) followed by Tukey multiple comparison test. $^{\$\$\$}p < 0.001$, $^{\$\$}p < 0.01$, $^{\$}p < 0.05$ compared to the sample without lysosomal inhibition.

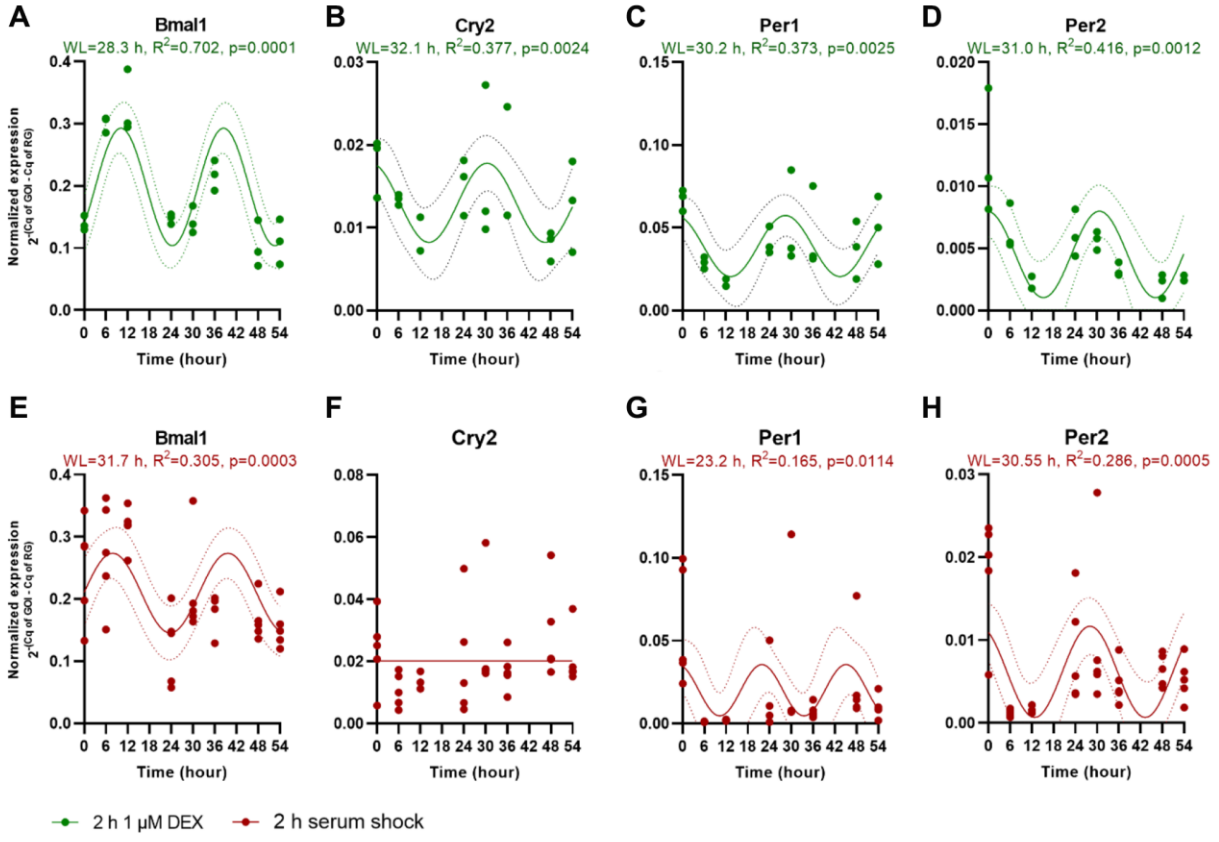

Supplementary Figure 5. mRNA levels of clock genes upon DEX treatment and serum shock in polarized ARPE-19 cells over a period of 54 hours. Normalized expression of (**A**) Bmal1, (**B**) Cry2, (**C**) Per1, (**D**) Per2 upon DEX treatment and (**E**) Bmal1, (**F**) Cry2, (**G**) Per1, (**H**) Per2 upon serum shock. Values are represented as scatter blot; *n* = 3–5. Sine waves with nonzero baseline + confidence bands were generated by comparison of fits (amplitude = 0 versus amplitude unconstrained). Abbreviation: WL: wavelength.

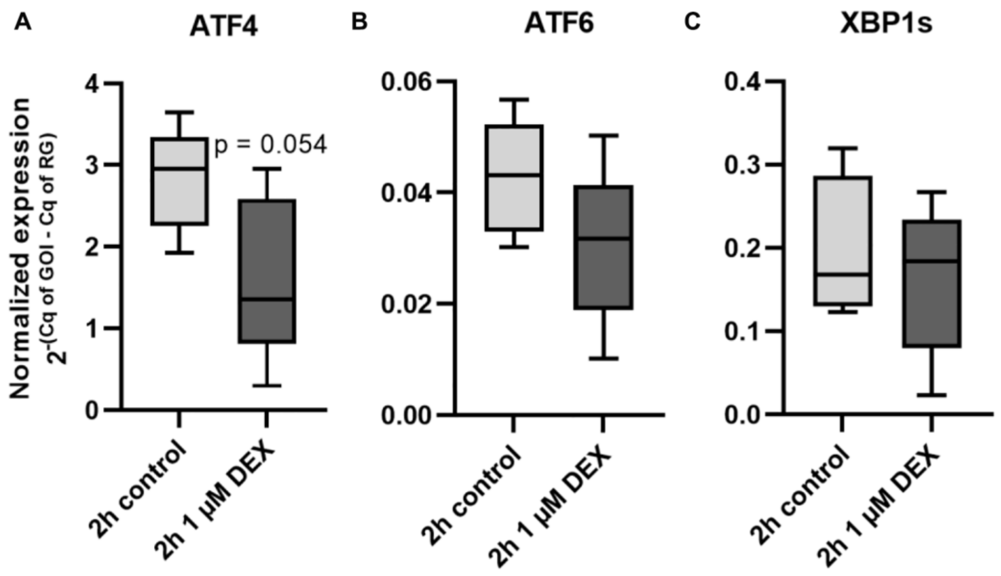

Supplementary Figure 6. UPR regulation upon dexamethasone treatment in polarized ARPE-19 cells. mRNA levels of (**A**) ATF4, (**B**) ATF6 and (**C**) XBP1s at 2 hours post dexamethasone (DEX) treatment in polarized ARPE-19 cells. Values are represented in box and whisker plot format (min to max); *n* = 5. The significance of differences upon dexamethasone treatment was calculated by a paired *t*-test.

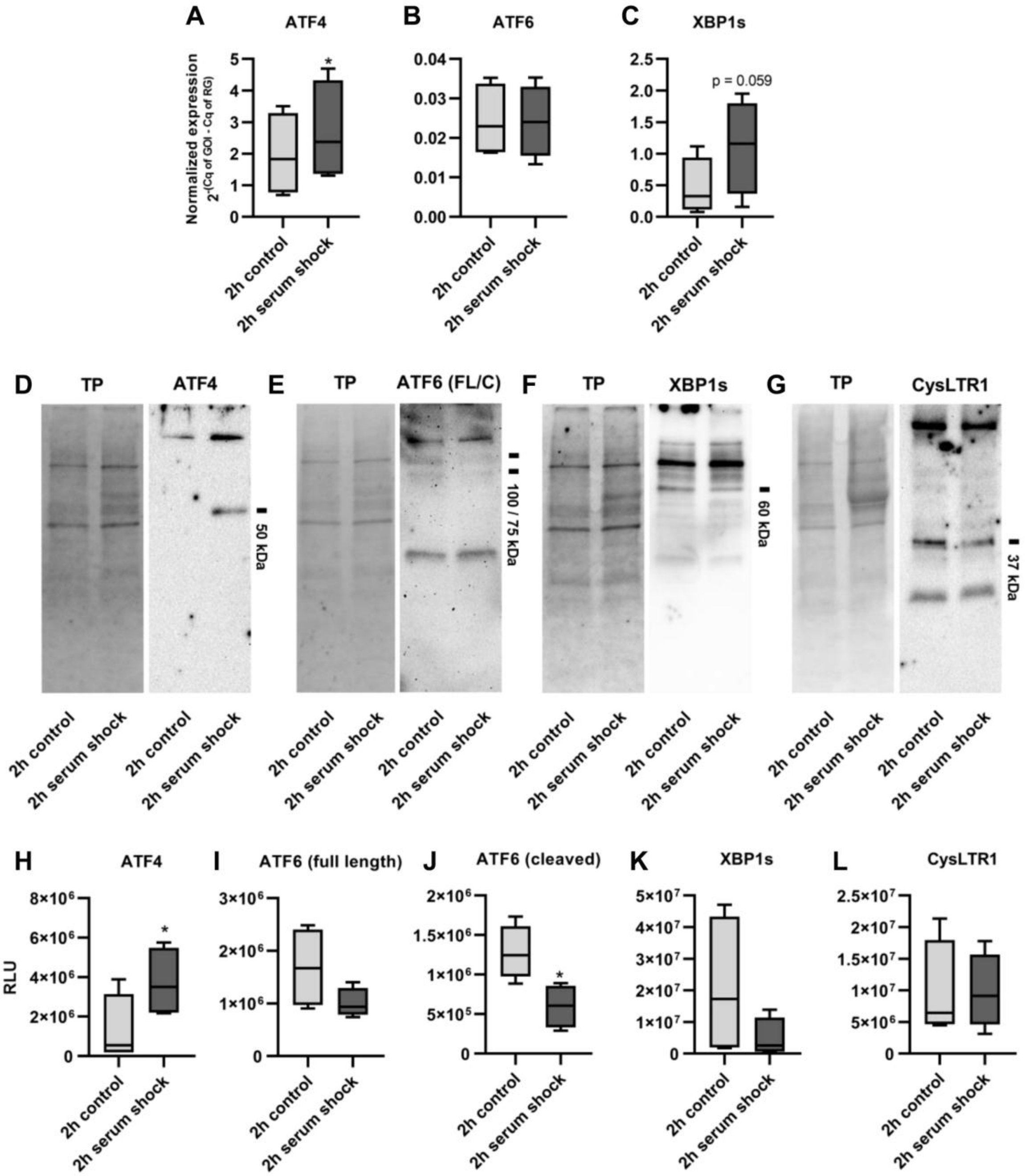

Supplementary Figure 7. UPR regulation upon serum shock in polarized ARPE-19 cells. mRNA levels of (**A**) ATF4, (**B**) ATF6 and (**C**) XBP1s at 2 hours post serum shock in polarized ARPE-19 cells. Values are represented in box and whisker plot format (min to max); $n = 4$. The significance of differences upon serum shock was calculated by a paired *t*-test. $^{*}p < 0.05$. Representative western blot analysis showing total protein (TP) loading and (**D**) ATF4, (**E**) ATF6, (**F**) XBP1s and (**G**) CysLTR1. RLU levels of (**H**) ATF4, (**I**) full-length ATF6, (**J**) cleaved ATF6, (**K**) XBP1s and (**L**) CysLTR1 normalized to the amount of total loaded protein in untreated and 2-hour serum shock-treated polarized ARPE-19 cells. Values are represented in box and whisker plot format (min to max); $n = 4$. The significance of differences upon serum shock was calculated by a paired *t*-test. $^{*}p < 0.05$.

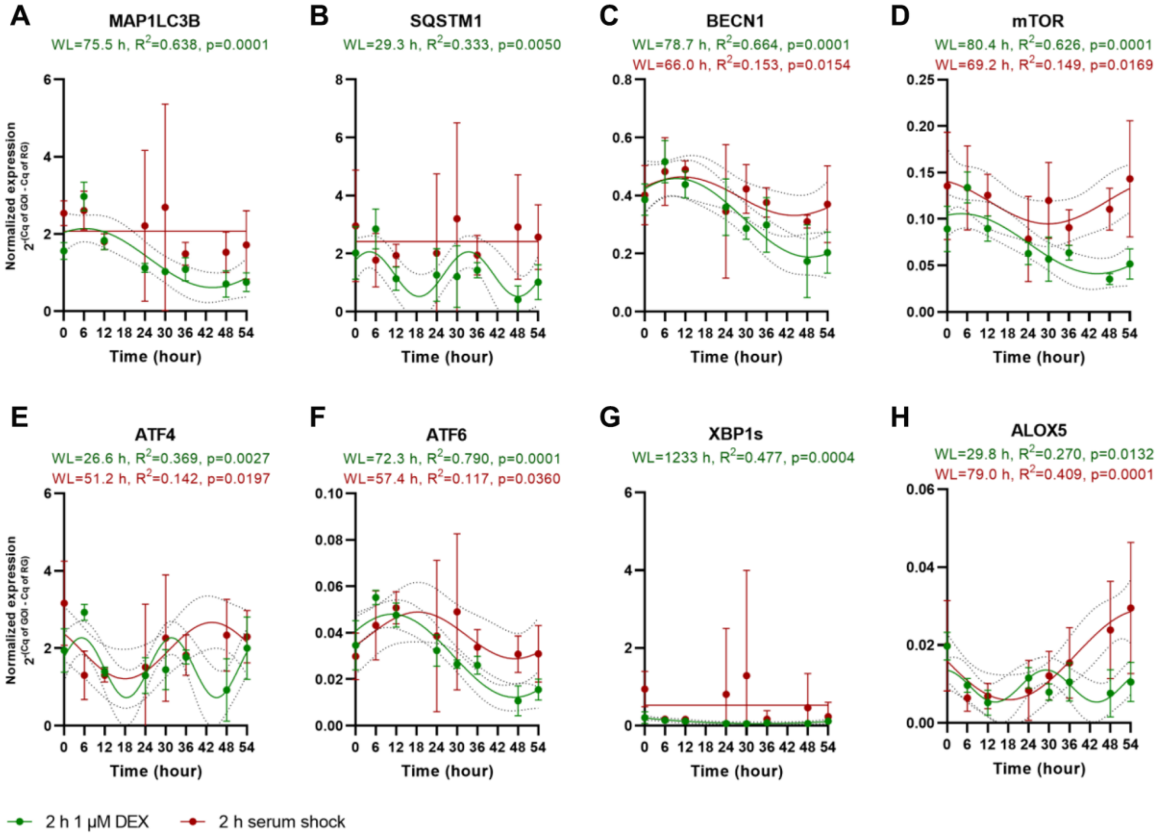

Supplementary Figure 8. mRNA levels of autophagic genes ((**A**) MAP1LC3B, (**B**), SQSTM1, (**C**) BECN1 and (**D**) mTOR), UPR transcription factors (**E**) ATF4, (**F**) ATF6 and (**G**) XBP1s and (**H**) ALOX5 upon DEX treatment and serum shock in polarized ARPE-19 cells over 54 hours. Values are represented as the mean ± SD; *n* = 3–5. Sine waves with nonzero baseline + confidence bands were generated with comparison of fits (amplitude = 0 versus amplitude unconstrained). Abbreviation: WL: wavelength.
